# A105 TRANSITION TO ADULTHOOD THROUGH COACHING AND EMPOWERMENT (TRACE): A FEASIBILITY RCT

**DOI:** 10.1093/jcag/gwae059.105

**Published:** 2025-02-10

**Authors:** K Beaudoin, H McKean, S Nsamenang, K Bortolin, T Cellucci, J Dowhaniuk, L Heale, R M Issenman, N Pai, M Sherlock, M Zachos, K Beattie, M Batthish, K Prowse

**Affiliations:** McMaster University, Hamilton, ON, Canada; McMaster Children’s Hospital, Hamilton, ON, Canada; McMaster Children’s Hospital, Hamilton, ON, Canada; McMaster Children’s Hospital, Hamilton, ON, Canada; McMaster Children’s Hospital, Hamilton, ON, Canada; McMaster Children’s Hospital, Hamilton, ON, Canada; McMaster Children’s Hospital, Hamilton, ON, Canada; McMaster Children’s Hospital, Hamilton, ON, Canada; McMaster Children’s Hospital, Hamilton, ON, Canada; McMaster Children’s Hospital, Hamilton, ON, Canada; McMaster Children’s Hospital, Hamilton, ON, Canada; McMaster Children’s Hospital, Hamilton, ON, Canada; McMaster Children’s Hospital, Hamilton, ON, Canada; McMaster Children’s Hospital, Hamilton, ON, Canada

## Abstract

**Background:**

Pediatric patients with chronic disease face challenges during the transition to adult care, including psychosocial and emotional stressors, as well as worsening disease.

**Aims:**

Transition to Adulthood through Coaching and Empowerment (TRACE) assessed the feasibility of conducting a virtual transition coaching randomized controlled trial for adolescent patients and collected preliminary clinical outcomes.

**Methods:**

Individuals aged 16-17 diagnosed with inflammatory bowel disease or juvenile idiopathic arthritis from the pediatric gastroenterology (GI) or rheumatology clinics at McMaster Children’s Hospital were recruited. Participants were randomized into control or Transition Coach Intervention (TCI) groups. TCI participants received 8 virtual coaching sessions; 6 with a child life specialist and 2 with a clinical psychologist. Electronic questionnaires were completed at baseline, 8 months, and 11 months. Feasibility outcomes, patient demographics, transition readiness, and quality of life were recorded. Descriptive statistics summarized feasibility outcomes and outcomes by group.

**Results:**

Of all participants (n=27), 67% were male, and the mean (SD) age was 16.8 (0.5) years. The recruitment rate was 58% and 38% for the GI and rheumatology clinics, respectively. The overall dropout rate was 7%. All TCI participants attended at least 6/8 sessions, and 63% of participants had a complete data set (Table I). Figure 1 shows baseline and 8-month follow-up data for preliminary clinical outcomes, such as transition readiness and quality of life, which will inform the design of a future randomized controlled trial.

**Conclusions:**

The TRACE study aims to show that a virtual TCI trial is feasible. Based on preliminary responses to this transition intervention, we plan to conduct a large-scale, randomized controlled trial.

Table I: Feasibility outcomes. (n=14 control group, n=13 TCI group, n=27 total). JIA = juvenile idiopathic arthritis, IBD = inflammatory bowel disease, TCI = transition coach intervention

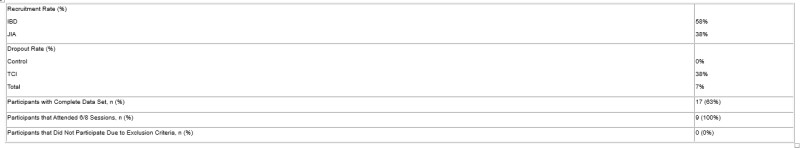

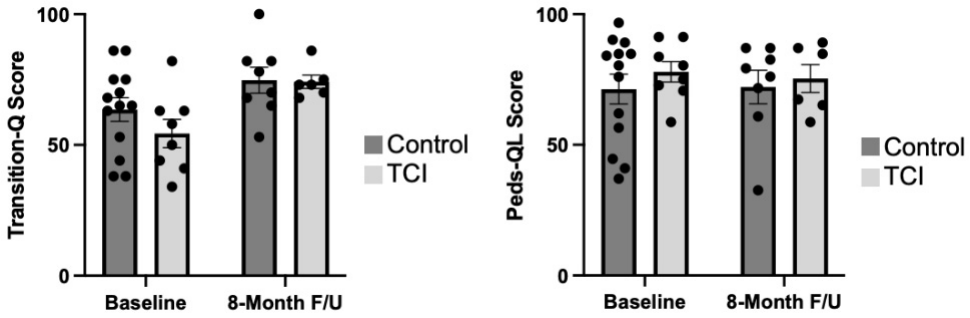

Figure 1. Transition-Q and Peds-QL scores at baseline and 8-month follow-up. (n=13 for the control group, n=8 for the baseline TCI group; n=8 for the 8-month follow-up control group, and n=6 for the 8-month follow-up TCI group). F/U = follow-up, TCI = transition coach intervention

Funding Agencies: HAHSO

